# Prognostic differences and implications on treatment strategies between butterfly glioblastoma and glioblastoma with unilateral corpus callosum infiltration

**DOI:** 10.1038/s41598-022-23794-6

**Published:** 2022-11-10

**Authors:** Mohammad Hazaymeh, Ronja Löber-Handwerker, Katja Döring, Tammam Abboud, Dorothee Mielke, Veit Rohde, Vesna Malinova

**Affiliations:** 1 Department of Neurosurgery, University Medical Center Göttingen, Georg-August-University, Robert-Koch-Straße 40, 37075 Göttingen, Germany; 2 Department of Neuroradiology, University Medical Center Göttingen, Göttingen, Germany

**Keywords:** Cancer imaging, Cancer

## Abstract

Approximately 25% of glioblastomas show at diagnosis a corpus callosum infiltration, which is associated with poor prognosis. The extent of corpus callosum involvement, however, ranges from partial unilateral to complete bilateral infiltration. The role of surgery in glioblastoma with corpus callosum involvement is controversial. In this study, we aimed to examine prognostic differences between glioblastoma with unilateral and glioblastoma with bilateral corpus callosum infiltration, and to evaluate possible treatment strategy implications. Patients with newly diagnosed glioblastoma from 2010 to 2019 were included. Corpus callosum infiltration was assessed in contrast-enhanced T1-weighted preoperative magnetic resonance imaging. Extent of resection, adjuvant treatments and overall survival were evaluated. Corpus callosum involvement was found in 96 (26.4%) out of 363 patients with newly diagnosed glioblastoma. Bilateral corpus callosum infiltration was found in 27 out of 96 patients (28%), and 69 patients had unilateral corpus callosum infiltration. Glioblastoma with corpus callosum affection had significantly lower median overall survival compared to glioblastoma without corpus callosum involvement (9 vs. 11 months, *p* = 0.02). A subgroup analysis of glioblastoma with unilateral corpus callosum infiltration revealed a significant difference in median overall survival dependent on extent of resection (6.5 without gross total resection vs. 11 months with gross total resection, Log-rank test *p* = 0.02). Our data confirms a shorter overall survival in glioblastoma subpopulation with corpus callosum involvement, especially for glioblastoma with bilateral corpus callosum infiltration. However, patients with partial corpus callosum infiltration undergoing gross total resection exhibited a significant survival benefit compared to their counterparts without gross total resection. Whenever reasonably achievable gross total resection should be considered as an integral part of the treatment strategy in glioblastoma with partial corpus callosum infiltration.

## Introduction

Glioblastoma is one of the most malignant brain tumors with an extremely aggressive behavior and treatment resistance resulting in a dismal prognosis with a very low chance of long-term survival^1^. The ability of tumor cells to migrate over long distances through brain tissue is one of the reasons why glioblastoma consistently recurs despite multimodal treatment including surgery and radio-chemotherapy. The tumor infiltration into key brain structures such as basal ganglia and brain stem is particularly associated with poor prognosis by causing neurological deficits and limiting a gross total resection of the tumor. Tumors affecting the corpus callosum are also expected to have a very limited overall survival because the corpus callosum is regarded as the route for tumor distribution to the contralateral hemisphere by tumor cell migration along the commissural fibers^2,3^. Gross total resection of tumors affecting the corpus callosum bears the risk for substantial neurocognitive deterioration with a reduction in quality of life and increased dependency during activities of daily life. Hence, there is a controversy in the current literature concerning the role of microsurgical resection of tumors affecting the corpus callosum^4–7^. In most centers, it is a common practice to perform only a tumor biopsy in patients with suspected glioblastoma with corpus callosum involvement on initial imaging followed by radio-chemotherapy^8^. However, the extent of corpus callosum infiltration may substantially vary between tumors, which could have an impact on survival as well. While most previously published series are focusing on tumors with bilateral corpus callosum infiltration^9,10^, the prognostic factors with potential treatment implications have not been sufficiently determined yet. Common limitations of previously published studies assessing the impact of surgical resection on survival in patients with glioblastoma affecting the corpus callosum are data selection, small number of included patients, and heterogenous treatment protocols preventing the authors from drawing general conclusions and from formulating treatment recommendations for management of glioblastoma affecting the corpus callosum. Furthermore, previously published studies on gliomas with corpus callosum involvement included low-grade and high-grade gliomas, while large patient series with glioblastoma involving the corpus callosum are rare. Another common shortcoming of previously published studies is the lack of clear differentiation between tumors primarily growing within the corpus callosum and lobar tumors with partial unilateral corpus callosum infiltration. A unilateral tumor infiltration of corpus callosum crossing the midline was already considered butterfly glioma in some of the studies^9^. The primary study objective was to assess survival differences of patients with glioblastoma affecting the corpus callosum dependent on the extent of corpus callosum involvement (unilateral vs. bilateral corpus callosum involvement), and to identify clinical, radiological, and molecular prognostic factors in this patient population. Additionally, we aimed to evaluate the impact of gross total resection on survival in patients with glioblastoma unilaterally infiltrating the corpus callosum.

## Materials and methods

### Patient population

A retrospective analysis of consecutive patients with newly diagnosed glioblastoma treated at our institution (Department of Neurosurgery, University Medical Center Göttingen) in the time-period between 2010 and 2019 was performed. Only adult patients with histologically confirmed glioblastoma according to the World Health Organization classification of tumors of the central nervous system (version 2016) were included in the study. Patients with secondary glioblastoma were excluded. The corpus callosum involvement was evaluated on the initial contrast-enhanced T1-weighted magnetic resonance imaging. Concerning the corpus callosum infiltration, the patients were divided into two groups: glioblastoma with unilateral corpus callosum included patients with unilateral tumor growth pattern and unilateral corpus callosum infiltration, and glioblastoma with bilateral corpus callosum infiltration encompassed the patients who had a bilateral growth pattern and bilateral corpus callosum Infiltration, so-called “butterfly glioblastoma”. Since no T2-weighted sequence was initially available in a large portion of the patient population, an involvement of corpus callosum on T2-weighted imaging could not be assessed in our patient cohort. The surgical treatment consisted of either gross total resection, subtotal resection, or biopsy. The extent of resection was assessed according to the early postoperative magnetic resonance imaging within 72 h of surgery. Gross total resection was achieved if an extent of resection of ≥ 95% of the tumor on contrast-enhanced T1-weighted sequence was documented on early postoperative magnetic resonance imaging. The decisions concerning the adjuvant treatment were made after a case discussion in the interdisciplinary conference (tumor board) for tumors of the central nervous system represented by following disciplines: neurosurgery, neurology, neuropathology, neuroradiology, oncology, and radiotherapy. Data on the initial Karnofsky Performance Status was gathered from the medical records. The applied adjuvant treatments are listed in Table 1. “Stupp completed” in Table 1 refers to patients who received radio-chemotherapy with concomitant temozolomide followed by completed six cycles chemotherapy with temozolomide as defined by Stupp et al. 2005^11^. In the contrary, “stupp partially completed” refers to patients, who received radio-chemotherapy with concomitant temozolomide and started cyclic chemotherapy with temozolomide but did not complete all six cycles due to side effects, clinical deterioration, or progression on imaging. A small proportion of patients was included in the GLARIUS trial and treated according to study protocol including Bevacizumab and Irinotecan^12^.

**Table 1:** Baseline parameters and tumor characteristics of the patient population.

	Glioblastoma with corpus callosum affection	Glioblastoma with unilateral corpus callosum infiltration	Glioblastoma with bilateral corpus callosum infiltration	*p* value
**Baseline parameters**				
Number of patients (%)	96 (100%)	69 (78%)	27 (22%)	
Mean age in years (SD)	62.1 (13.9)	62.7 (13.2)	60.5 (15.6)	0.49
**Sex**				
Male	55/96 (57%)	39/69 (57%)	16/27 (59%)	0.80
Female	41/96 (43%)	30/69 (43%)	11/27 (41%)	
Mean initial KPS (SD)	77.4% (13.5)	77.7% (13.5)	76.7% (13.6)	0.74
**Tumor characteristics**				
**Tumor location within corpus callosum**				
Anterior	43/96 (45%)	32/69 (46%)	11/27 (41%)	0.82
Middle	13/96 (14%)	8/69 (12%)	5/27 (18%)	0.50
Posterior	40/96 (41%)	29/69 (42%)	11/27 (41%)	0.99
**Mean tumor volume in ml (SD)**				
Overall	42.9 (31.6)	45.5 (32.1)	36.2 (29.7)	0.15
Tumor within corpus callosum	6.4 (8.9)	3 (2.6)	15.3 (12.5)	< 0.0001*
Tumor outside corpus callosum	36.5 (30.7)	42.5 (31.3)	20.9 (23)	0.007*
**Molecular markers**				
IDH mutation	8/66 (12%)	3/50 (6%)	5/16 (31%)	0.01*
MGMT methylation	30/66 (45%)	24/50 (48%)	6/16 (38%)	0.56
Positive p53	41/66 (62%)	30/50 (60%)	11/16 (69%)	0.57
Positive Olig2	52/66 (79%)	38/50 (76%)	14/16 (87%)	0.48
Mean Ki67% (SD)	12.5 (10.2)	11.3 (6.9)	16.1 (16.1)	0.82
Missing values	20/96 (21%)	19/69 (17%)	11/27 (41%)	0.22

*KPS* Karnofsky Performance Status, *SD* standard deviation, *IDH* Isocitrate dehydrogenase, *MGMT* O6-methylguanine-DNA methyltransferase-methylation, *p53* transcription factor and tumor suppression gene, *Olig2* transcription factor and stem cell marker, *Ki67* nuclear protein and tumor cell proliferation marker.

A p value < 0.05 was considered statistically significant.

The significant parameters are marked with *.

### Tumor characteristics

Volumetric analysis of overall tumor volume as well as tumor volume within the corpus callosum was performed using a volume contrast-enhanced T1-weighted dataset and by means of the Brainlab® software (Brainlab AG, Munich, Germany) by a single examiner (M.H.). Additionally, the tumor location within the corpus callosum (anterior, middle, posterior) was documented. In patients, who initially underwent a gross total resection, the timepoint of tumor recurrence and the location of the recurrent tumor were additionally examined on the follow-up magnetic resonance imaging scans. The examination of specific molecular markers of the tumor has been conducted at our institution on a regular basis since 2016, which is why molecular markers were only available for patients diagnosed in 2016 or later. The following molecular parameters were assessed: MGMT (O6-methylguanine-DNA methyltransferase)-methylation, IDH (isocitrate dehydrogenase) mutation, transcription factor and tumor suppression gene (p53), transcription factor and stem cell marker (Olig2), nuclear protein and tumor cell proliferation marker (Ki67). The patient proportion without molecular markers were stated as missing values in Table 1.

### Outcome parameters

The primary outcome parameter was overall survival defined as the time from glioblastoma diagnosis to the time of death. Data on the course of disease and the date of death were extracted from the medical records and the electronical documentation system of our institution. The prognostic value of clinical, radiological, and molecular tumor-specific markers was evaluated. The impact of gross total resection on survival was assessed in a subgroup analysis of glioblastoma with unilateral corpus callosum infiltration. Additionally, the progression free survival was evaluated according to the response assessment in neuro-oncology criteria (RANO) at 6-months follow-up imaging^13^.

### Statistical analysis

The statistical analysis was performed using the GraphPad Prism statistics software (Version 9, GraphPad Software, San Diego, CA, USA). Descriptive statistics was applied for the depiction of baseline characteristics. Kaplan Meier survival curves were generated for the assessment and comparison of overall survival between groups. ANOVA analysis was done for comparison of more than two groups. Multivariate logistic regression analysis was performed to identify independent predictors of longer overall survival. A *p* value of < 0.05 was considered statistically significant.

### Ethical approval

The study was approved by the local ethics committee of the University Medicine Göttingen (Study identification number 16/12/20). Due to retrospective nature of the study informed consent was deemed not necessary and all the procedures being performed were part of the routine care. All procedures performed in this study were in accordance with the ethical standards of institutional and/ or national research committee and with the 1964 Helsinki Declaration.

### Consent to participate

Due to retrospective nature of the study informed consent was waived by the Ethics Committee of the University Medical Center Göttingen (Chair Prof. Dr. Jürgen Brockmöller).

## Results

### Patient population

Between 2010 and 2019, 363 consecutive patients with newly diagnosed glioblastoma were treated at our institution. Ninety-six (26.4%) of these patients were identified to have tumors involving the corpus callosum and were considered for further analysis of the study. Regarding the corpus callosum involvement, 69 patients (78%) presented with unilateral tumor growth pattern and unilateral corpus callosum infiltration, whereby 27 patients (22%) had tumors with bilateral growth pattern and bilateral infiltration of CC. The baseline characteristics of the patient population are summarized in Table 1 showing no statistically significant differences between both patient groups.

### Tumor characteristics

A detailed overview of tumor parameters for both groups is given in Table 1. There were no significant differences between both groups concerning the tumor location within corpus callosum (anterior, middle, or posterior corpus callosum). The mean tumor volume within the corpus callosum was significantly lower in glioblastoma with unilateral corpus callosum infiltration compared to glioblastoma with bilateral corpus callosum infiltration. On the contrary, the mean overall tumor volume did not significantly differ between both groups. While the proportion of patients with isocitrate dehydrogenase mutation was significantly higher in glioblastoma with bilateral corpus callosum infiltration compared to glioblastoma with unilateral corpus callosum infiltration, no significant differences could be found for the distribution of the remaining molecular markers in both patient groups.

### Surgical and adjuvant treatment

**Figure 1:**
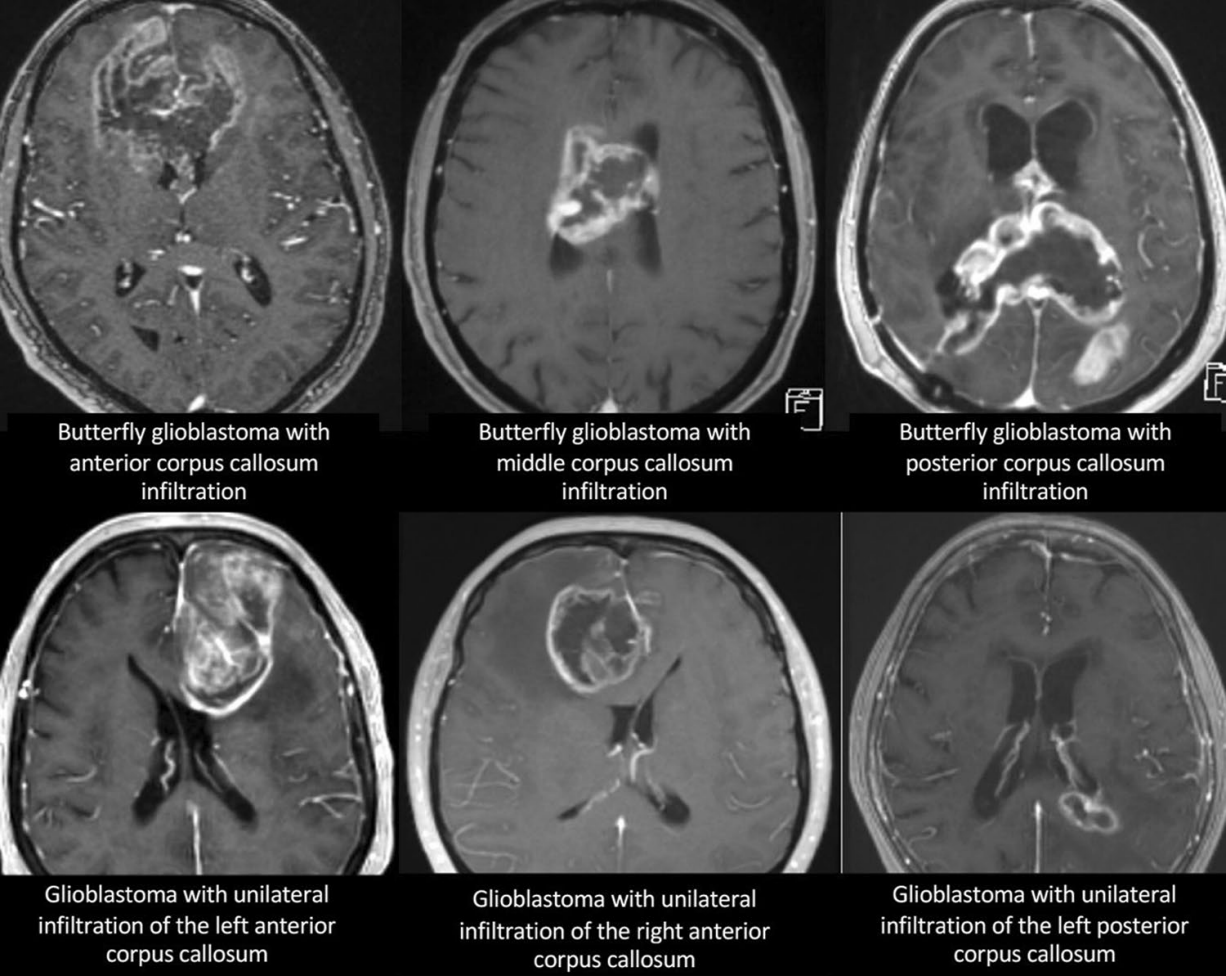
Examples of glioblastoma with unilateral corpus callosum infiltration and butterfly glioblastoma with bilateral corpus callosum infiltration scheduled to biopsy and gross total resection, respectively.

Table 2 summarizes the data on surgical and adjuvant treatments in the patient groups. Most patients received adjuvant treatment according to Stupp protocol, which was comparable between both groups (67% vs. 63%). The proportion of patients, who completed the treatment according to the Stupp protocol did not significantly differ between both groups (41% vs. 37%). In 10% of all patients a treatment according to the Stupp protocol was recommended but no information was available whether the treatment was indeed carried out or not due to lost to follow-up. The surgical approach differed significantly between glioblastoma with unilateral corpus callosum infiltration and glioblastoma with bilateral corpus callosum infiltration with larger proportion of biopsy in glioblastoma with bilateral corpus callosum infiltration compared to glioblastoma with unilateral corpus callosum infiltration, and gross total resection only performed in glioblastoma with unilateral corpus callosum infiltration. Gross total resection was performed in patients either with infiltration of the anterior corpus callosum (54%, 15/28) or the posterior corpus callosum (46%, 13/28). An infiltration of the middle corpus callosum was considered unsuitable for resection due to eloquent location. Examples of different tumor locations and surgical decisions are given in Fig. 1. At 6-month follow-up a tumor recurrence was found in 67.8% of the patients, who initially underwent tumor gross total resection, of whom 47.3% had a local recurrence at the edge of the former resection cavity, 42.2% developed a distant recurrence, and 10.5% had a recurrence within the contralateral corpus callosum.

**Table 2:** Surgical and adjuvant tumor treatment.

	Glioblastoma with corpus callosum affection	Glioblastoma with unilateral corpus callosum infiltration	Glioblastoma with bilateral corpus callosum infiltration	*p* value
Number of patients	96	69	27	
**Extent of surgery**				
Gross total resection	28/96 (29%)	28/69 (41%)	0/27 (0%)	0.0001*
Subtotal resection	19/96 (18%)	16/69 (23%)	3/27 (11%)	0.25
Biopsy	49/96 (51%)	25/69 (36%)	24/27 (89%)	< 0.0001*
**Adjuvant treatment**				
Stupp completed	38/96 (40%)	28/69 (41%)	10/27 (37%)	0.81
Stupp partially completed	25/96 (26%)	18/69 (26%)	7/27 (26%)	0.98
Stupp recommended	10/96 (10%)	5/69 (7%)	5/27 (19%)	0.13
Bevacizumab + Irinotecan	3/96 (3%)	2/69 (10%)	1/27 (4%)	0.84
No treatment	12/96 (13%)	9/69 (13%)	3/27 (11%)	0.79

A p value < 0.05 was considered statistically significant.

The significant parameters are marked with *.

### Survival analysis

The patients presenting with glioblastoma affecting the corpus callosum (96 patients) had a median overall survival of 9 months (95% CI 6–11), that was significantly lower compared to median overall survival (11 months, 95% CI 9–12) of the consecutive glioblastoma cohort (267 patients) without corpus callosum involvement (*p* = 0.02). The median overall survival in glioblastoma with unilateral corpus callosum infiltration (10 months, 95% CI 6–11) was higher compared to glioblastoma with bilateral corpus callosum infiltration (7 months, 95% CI 2–11), but the difference was not significant (*p* = 0.52). There was a trend to lower median overall survival in the patient group with unilateral corpus callosum involvement compared to the consecutive glioblastoma cohort without corpus callosum affection (10 vs. 11 months, *p* = 0.07). The proportion of patients with overall survival longer than 12 months in glioblastoma with bilateral corpus callosum infiltration was lower compared to glioblastoma with unilateral corpus callosum infiltration (18 vs. 25%) but the difference was not significant (*p* = 0.26).

**Figure 2:**
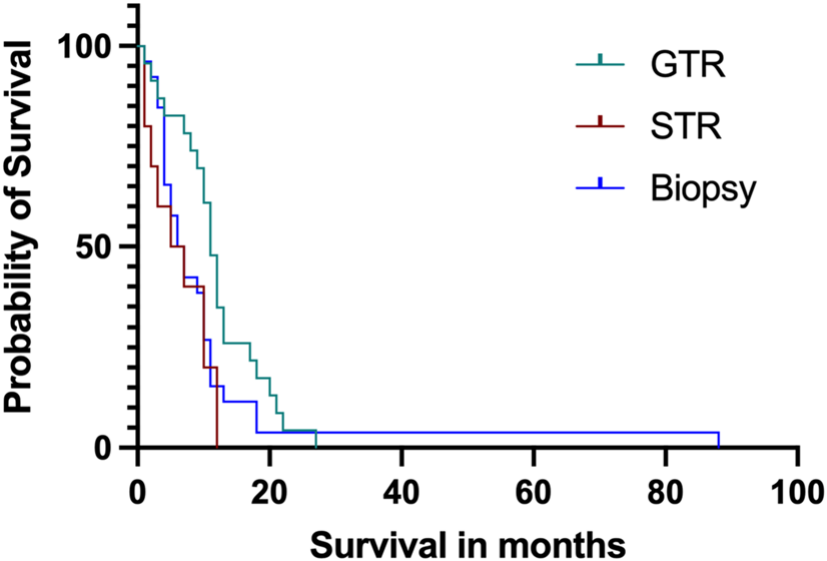
Kaplan Meier survival curve of the patient group with unilateral corpus callosum infiltration showing a significant difference in overall survival dependent on the extent of resection Log-rank (Mantel-Cox) test *p* = 0.02 (median overall survival in patients without gross total resection 6.5 vs. 11 months in patients with gross total resection). GTR = gross total resection, STR = subtotal resection.

A subgroup survival analysis of glioblastoma with unilateral corpus callosum infiltration revealed the following results. Kaplan Meier survival curve (Fig. 2) of glioblastoma with unilateral corpus callosum infiltration showed a significant difference in overall survival dependent on the extent of resection (median overall survival in glioblastoma with unilateral corpus callosum infiltration without gross total resection 6.5 [95% CI 4–10] versus 11 [95% CI 9–13] months with gross total resection, Log-rank (Mantel-Cox) test, Chi square 4.153, Hazard ratio Log-rank 0.6045, 95% CI 0.3556 to 1.027, *p* = 0.02). In the univariate subgroup analysis of patients with unilateral corpus callosum infiltration an overall survival of longer than 12 months significantly correlated with younger age at diagnosis (r = − 0.3696, *p* = 0.004), higher initial Karnofsky performance status (r = 0.2787, *p* = 0.03), gross total resection (r = 0.2563, *p* = 0.04), and with completed Stupp protocol (r = 0.2957, *p* = 0.02). No correlation was found between overall survival and tumor volume or tumor location within corpus callosum. In the multivariate logistic regression analysis in glioblastoma with bilateral corpus callosum infiltration none of the parameters (age at diagnosis, initial Karnofsky performance status, gross total resection and completed Stupp protocol) was an independent predictor of overall survival longer than 12 months. The subgroup analysis in glioblastoma with bilateral corpus callosum infiltration revealed no prognostic factors associated with overall survival of longer than 12 months. Subtotal resection was not associated with longer overall survival, neither in glioblastoma with unilateral corpus callosum infiltration (r = − 01758, (*p* = 0.19) nor in glioblastoma with bilateral corpus callosum infiltration (r = − 0.1690, *p* = 0.51).

## Discussion

The primary goal of this observational study was to evaluate survival differences in a subpopulation of consecutive patients with newly diagnosed glioblastoma affecting the corpus callosum stratified for the extent of corpus callosum involvement (unilateral vs. bilateral corpus callosum infiltration). A corpus callosum affection was found in approximately one quarter of consecutive GBM patients, of whom two-thirds were unilaterally infiltrating the corpus callosum and one-third of the patients were bearing tumors with a bilateral distribution within the corpus callosum. Our study confirmed a significantly lower overall survival in patients with glioblastoma involving the corpus callosum compared to their counterparts without corpus callosum involvement, which is in line with the results of a recently published study by Cui et al.^14^. While overall survival of patients with glioblastoma unilaterally infiltrating the corpus callosum was comparable to the overall survival in the patient cohort without corpus callosum affection, patients with bilateral corpus callosum infiltration experienced a significantly lower overall survival. The patient cohort with unilateral corpus callosum affection was found to have similar prognostic factors (younger age at diagnosis, higher initial Karnofsky performance status) as the patient cohort without corpus callosum involvement. In the contrary, no specific prognostic factors could be identified in the patient group with tumors bilaterally infiltration the corpus callosum. Gross total resection and completed adjuvant treatment according to the Stupp protocol were found to be associated with longer overall survival of at least 12 months in the patient group with unilateral corpus callosum infiltration. The findings of our study are implying that glioblastoma patients with unilateral corpus callosum infiltration could be treated with a similar strategy to GBMs with singular tumor manifestation on imaging without corpus callosum involvement. According to the findings of our study, gross total resection whenever reasonably achievable should be considered as an integral part of the treatment strategy in glioblastoma with partial corpus callosum infiltration. Since gross total resection was not conducted in patients with glioblastoma bilaterally growing within the corpus callosum, our study cannot provide an answer to the question whether gross total resection might be reasonable in this patient subpopulation. Subtotal resection resulted in no survival benefit neither in the patient group with unilateral nor in the patient group with bilateral corpus callosum infiltration.

### Involvement of corpus callosum as a prognostic factor in glioblastoma patients

The involvement of the corpus callosum is considered a negative prognostic factor in glioblastoma patients^2,15^. A recently published study by Fyllingen et al. demonstrated a correlation of glioblastoma infiltration of key brain structures including the corpus callosum with an overall survival of less than 6 months^16^. Additionally, Mistry et al. found an increased frequency of multifocality in glioblastoma with corpus callosum involvement, which may also contribute to a poor prognosis in these patients^3^. A generally accepted theory of brain tumor development is that tumors tend to grow along blood vessels or white matter fibers, which serve as a route for cancer cell migration^17,18^. Thus, tumors involving white matter tracts have direct infiltration routes to other distant areas of the brain which often results in limited treatment options and decreased survival^15^. However, corpus callosum infiltration was associated with shorter survival only in the univariate analysis, while the multivariate analysis revealed a contact with the subventricular zone as an independent predictor of shorter overall survival in the study of Mistry et al.^3^. Although, glioblastoma with corpus callosum involvement had also a contact with subventricular zone in 92% of this study, corpus callosum infiltration was not an independent survival predictor. The authors have interpreted this finding as rather coincidental due to a vicinity of corpus callosum to the ventricular system^3^. According to this, multiple factors seem to co-influence the prognostic value of corpus callosum infiltration, that should be considered. On a molecular level, Cui et al. found that glioblastoma with corpus callosum affection tend to have a higher incidence of platelet derived growth factor receptor alpha alterations than glioblastoma without corpus callosum involvement which in turn is associated with worse survival. Furthermore, patients with glioblastoma affecting the corpus callosum had a lower extent of resection rates than their counterparts with glioblastoma without corpus callosum infiltration^19^. For these reasons, tumors with corpus callosum involvement are more often considered for biopsy than for resection, which is probably at least partly responsible for the worse overall survival in this patient population. A tumor resection in these patients is mostly deemed futile because a rapid recurrence within the corpus callosum or on the contralateral hemisphere is expected to occur after resection of glioblastoma with corpus callosum affection. However, the extent of corpus callosum involvement may substantially vary, which would imply a need for different treatment strategy for patients with unilateral and bilateral corpus callosum affection, which is supported by the findings of our study. Gross total resection was conducted in the half of patients with unilateral corpus callosum infiltration in our study, that resulted in significantly higher overall survival. Concerning the recurrence pattern only a small proportion (11%) of patients with gross total resection had a recurrence within the contralateral corpus callosum. However, a higher proportion of patients presented with distant recurrence on both hemisphere, which is higher compared to the reported overall incidence of distant recurrence in glioblastoma patients of approximately 20%, which is in line with the reported higher rate of multifocality in glioblastoma involving the corpus callosum.

### Survival benefit of surgical resection of glioblastoma with corpus callosum involvement

Several retrospective series evaluating the benefit of surgical resection of gliomas involving the corpus callosum has been previously published^2,7,9,10,13,20,21^. Most studies are focusing on glioblastoma with bilateral corpus callosum infiltration, whereas studies evaluating glioblastoma with unilateral corpus callosum infiltration are underrepresented in the literature. Chaichana et al. performed a matched-pair analysis of patients with butterfly glioblastoma comparing 11 patients undergoing tumor debulking with 11 patients receiving tumor biopsy and found a significantly longer survival in the patient group with tumor resection (7 vs. 3.5 months median overall survival). An extent of resection of more than 65%, radiation and chemotherapy with temozolomide were associated with longer survival in the patient group undergoing tumor resection^2^. Similar results were published by Opoku-Darko et al., who reported a higher survival in 29 patients with butterfly glioblastoma with tumor resection (50% of patients with > 98% extent of resection) compared to biopsied patients (7.8 vs. 2.8 months). However, adjuvant treatment received most patients after tumor resection and only the half of biopsied patients, which might have had influenced the results in this study^22^. Dayani et al. have assessed the impact of tumor resection on survival in a series of 39 patients with butterfly glioblastoma invading both hemispheres and the corpus callosum, of whom 14 patients had tumor resection and 25 patients received a biopsy. The reported mean extent of resection was 81.7%, whereas a minimum extent of resection of 86% was associated with survival benefit^10^. However, only the half of patients undergoing biopsy received tumor treatment afterwards. Additionally, the patient cohort encompassed patients treated before 2005, hence, before the Stupp protocol has become a standard treatment for glioblastoma patients. Thus, most patients received mainly radiotherapy as adjuvant treatment^10^. The patient group with butterfly glioblastoma in our study involved 27 patients, of whom 24 patients received adjuvant treatment and only 3 patients had no treatment after biopsy. Most patients in our study were treated according to Stupp protocol. These differences may be the explanation for the longer overall survival found in biopsied butterfly glioblastoma in our study compared to previously published patient series (median overall survival 7 vs. 3 months). In contrast to previous series, subtotal resection had no impact on survival in the patient groups included in our study. Baoro et al. reported one of the largest series with butterfly glioblastoma including 62 patients treated in the time-period 2008 and 2018 with surgical resection (median extent of resection of 72.3%) in 26 patients and biopsy in 36 patients. The median overall survival in this study was 8.7 month which was in line with the median overall survival in our patient cohort^21^. A recently published study including 55 isocitrate-dehydrogenase-wild type glioma patients with uni- or bilateral corpus callosum infiltration treated in the time-period between 2005 and 2017 demonstrated an increase of 2-year survival rate from 7% of biopsied patients to 30% of patients with tumor resection. However, only patients with gross total resection had still a survival benefit in the multivariate analysis, that could be achieved in only 30% of operated patients^20^. Furthermore, the authors did not differentiate between unilateral and bilateral corpus callosum infiltration, not allowing a direct comparison with the gross total resection rate in our study, since gross total resection was performed only in the patient group with unilateral corpus callosum infiltration in our study cohort. To the best of our knowledge, our study included the largest series of glioblastoma with unilaterally corpus callosum infiltration demonstrated a survival benefit of gross total resection, that was performed in 41% of cases, resulting into a median overall survival of 11 months. Considering the results of previously published studies and of our study, surgical resection contributes to a survival benefit in glioblastoma with corpus callosum affection. A limitation of our study and previously published retrospective studies is the missing data on neurocognitive function before and after tumor resection, which is of high relevance for the preservation of quality of live in this patient population. This question was addressed in a recently published prospective study. Forster et al. have evaluated possible benefits of surgical resection in patients with gliomas involving the corpus callosum and investigated its effect on neurocognition. The cohort included 21 patients with varying degrees of corpus callosum involvement and different histological diagnosis (17 patients with glioblastoma, 2 with oligodendroglioma, one patient with low grade diffuse astrocytoma, and one patient with anaplastic astrocytoma). A complete tumor resection was achieved in 15 patients, whereas 6 patients had a subtotal tumor removal. A decline in neurocognition was observed within the first postoperative days, which dramatically improved in every neurocognition domain after at least 3.1 months^7^. The risk for neurocognitive decline with substantial reduction in quality of life on the one side needs to be weighed up against the possible survival benefit of gross total resection in patients with glioblastoma affecting the corpus callosum. A recently published study by Cui et al. have demonstrated a benefit of using multimodal intraoperative techniques such as neuronavigation, intraoperative monitoring, and intraoperative magnetic resonance imaging for resection of tumors with uni- or bilateral corpus callosum infiltration. The multimodal group had a higher median extent of resection as well as rate of gross total resection than the conventional group. The survival analysis demonstrated that the multimodal group had a longer median progression free survival (9.5 vs. 7.0 months) and overall survival (15.9 vs. 11.6 months) compared to the conventional group^13^. Furthermore, the establishment of prognostic factors for a reliable patient selection for tumor resection would facilitate the identification of patients with survival benefit from surgical resection. While already established prognostic factors like younger patient age and better clinical condition could be confirmed as prognostic factors in glioblastoma with unilateral corpus callosum infiltration of our study, this did not apply for glioblastoma with bilateral corpus callosum infiltration.

### Limitations of the study

Limitations of our study include its retrospective nature, and consecutively the bias in deciding which patients should undergo a resection or biopsy. Furthermore, analysis of molecular tumor markers started in 2016 at our hospital, which is why there is a large amount of missing data, which in turn limited further analysis of this aspect. Additionally, data on the neuropsychological performance of the patients was not available in our retrospective patient cohort, which is another limitation of the study. Future prospective study with pre- and postoperative neuropsychological evaluation is needed to evaluate possible impact of gross total resection on the neuropsychological performance of the operated patients.

## Conclusion

Our data confirms a shorter overall survival in glioblastoma subpopulation presenting with corpus callosum involvement, especially for glioblastoma with butterfly growth pattern. However, patients with partial unilateral corpus callosum infiltration undergoing gross total resection exhibited a significant survival benefit compared to their counterparts without gross total resection. Whenever reasonably achievable gross total resection should be considered as an integral part of the treatment strategy in glioblastoma with partial infiltration of the anterior or posterior corpus callosum.

## Data Availability

All available data is presented in the manuscript.
